# A needle in the haystack: Single-cell omics of the distinct xylem differentiation programs in gymnosperms and angiosperms

**DOI:** 10.1093/plcell/koaf262

**Published:** 2025-11-05

**Authors:** Leonard Blaschek

**Affiliations:** Assistant Features Editor, The Plant Cell, American Society of Plant Biologists; Department of Plant & Environmental Sciences, University of Copenhagen, Frederiksberg C 1871, Denmark

Disentangling the cellular differentiation programs in the xylem—a tissue responsible for structural support and long-distance water transport—has proved a formidable challenge for decades. Xylem differentiation is a highly convoluted process containing various cell types at all stages of their differentiation programs at any given time. Isolating the distinct developmental stages of a specific cell type for bulk analysis of their transcriptome, proteome, or metabolome is nearly impossible. Previous attempts to make xylem cells more amenable to investigation with traditional tools have focused on forcing synchronicity onto the system. Inducible overexpression of xylem master regulators VND6 or VND7 triggers trans-differentiation of all cells in Arabidopsis seedlings into xylem vessel elements (Yamaguchi et al. 2010). Alternatively, Arabidopsis stem cell cultures can be induced with a combination of plant hormones to trigger synchronous differentiation of xylem cells in suspension (Derbyshire et al. 2015). Both systems have catalyzed crucial advances in our understanding of xylem formation. However, they create artificial environments, lacking native tissue contexts and cell proportions—key aspects for the interconnected xylem.

In new work, Peng Shuai and colleagues (Shuai et al. 2025) used integrated single-cell transcriptomics, proteomics, and metabolomics with spatial information to follow the differentiation of xylem cells in the gymnosperm *Cunninghamia lanceolata*. By placing their results in the context of previously published single-cell transcriptomics data from 4 angiosperms (Tung et al. 2023), the authors finely mapped the similarities and differences in xylem differentiation programs between the 2 primary clades of seed plants, angiosperms and gymnosperms. In angiosperms, xylem cells formed several lineages, terminating in 4 clusters of fully differentiated cells: vessels, rays, and 2 groups of libriform fibers. The vessel cluster also included the tracheids of the angiosperm *Trochodendron aralioides*, which underwent an evolutionary reversal toward gymnosperm-like xylem architecture. Generating gene expression profiles for 7009 individual *C. lanceolata* xylem cells, the authors recovered all clusters previously identified in angiosperms. Since none of the 4 tested angiosperms covered all clusters (magnoliids and eudicots had distinct types of initials and fibers), this meant that gymnosperms seemed to exhibit more transcriptional variation in their xylem than angiosperms (Fig.). Indeed, despite their morphological homogeneity, gymnosperm tracheids were comprised of subclusters resembling both angiosperm vessels and libriform fibers, as well as a cluster that was completely absent in angiosperms. Using lineage tracing, this last cluster was determined to emerge from the vessel-like cluster, thereby representing an additional, later stage in the vessel-like lineage, perhaps reflecting the longer differentiation time of tracheids compared to vessels (Bollhöner et al. 2012). Finally, to confirm their cluster assignments, the authors also performed in situ transcriptomics and metabolomics, as well as laser-dissection-based proteomics, correlating protein and lignin precursor levels with the corresponding transcriptomes.

**Figure.**
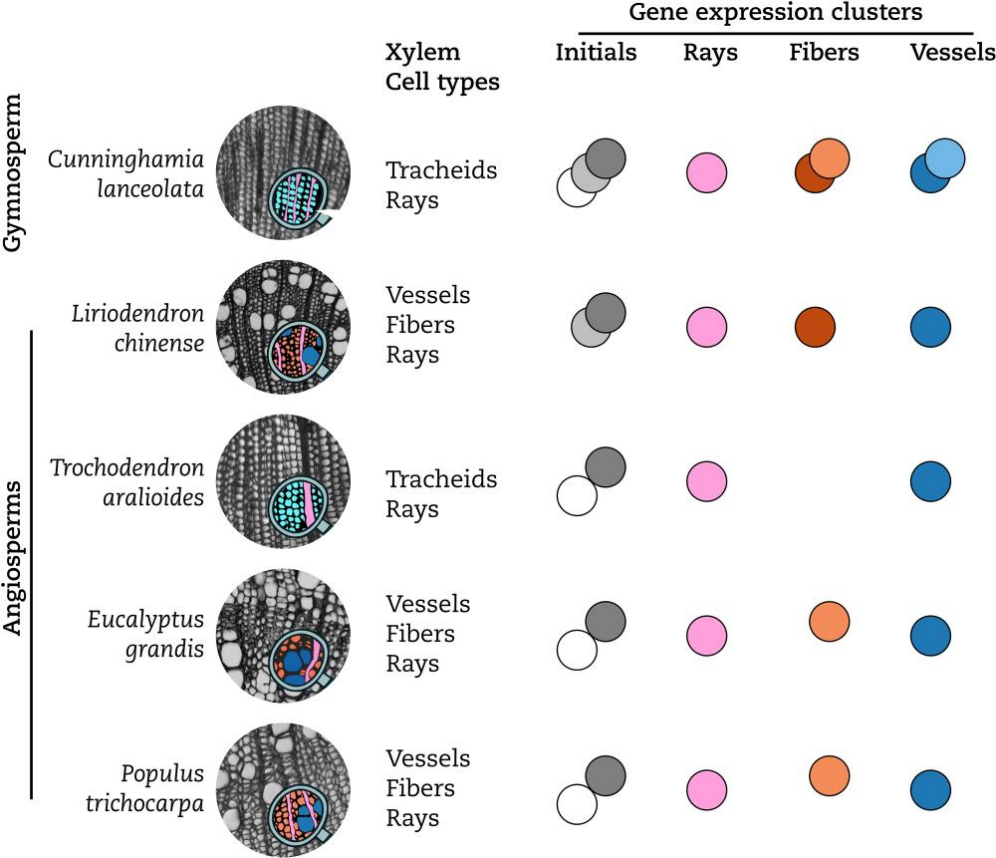
Morphological xylem cell types and single-cell gene expression clusters observed in the 5 analyzed species. Despite its morphological homogeneity, the gymnosperm *C. lanceolata* is the only tested species that contains cells covering the whole range of observed transcriptional variety. Figure adapted from Shuai et al. (2025), Figures 1 and 15.

Shuai et al. (2025) provide insights into the transcriptional dynamics of single, differentiating xylem cells. The extent of morphologically invisible transcriptional variation is striking, sparking numerous follow-up questions. How, for example, would the xylem of the gnetophytes, a group of vessel-forming gymnosperms, fit into this landscape? There are also some conflicts with our previous understanding of the xylem that need to be resolved. Perhaps the most surprising is that single-cell transcriptomics in *Populus trichocarpa* identified 0.38 fibers for every vessel (433:1140 cells), while morphological quantification in the xylem of trees in the same greenhouse identified 21 fibers for every vessel (2255:105 cells). This overestimation of vessel numbers harks back to the observed variation in gymnosperm tracheids, where morphologically indistinguishable cells would diverge dramatically in their transcriptional profiles, clustering with either angiosperm vessels or libriform fibers. It is possible that the angiosperm xylem contains a similar, morphologically invisible axis of transcriptional variation, which places a large subpopulation of libriform fibers into the vessel cluster. However, whether this transcriptional variation results in real, functional differences or is overwritten by, for example, post-translational regulation remains to be tested. Additionally, the recovered cluster sizes, combined with the observation that only the libriform fiber cluster expressed significant amounts of monolignol biosynthesis genes, would suggest that as little as 10% of xylem cells were responsible for the entirety of monolignol biosynthesis. This stands in apparent contrast to previous approaches using cell cultures and VND7 overexpressing seedlings, which actively express monolignol biosynthesis genes while being devoid of libriform fibers (Li et al. 2016; Blaschek et al. 2023). This contrast, however, might also simply stem from differences in species or tissue type, with both Arabidopsis systems representing primary, not secondary, xylem.

Despite, or perhaps rather because of, the numerous remaining questions, the data produced by Shuai et al. (2025) represent an immensely valuable and exciting resource for future work on the physiology and evolution of the xylem.

## Recent related articles in *The Plant Cell*:

- Grones et al. (2024) discussed best practices for scRNA-seq experiments.
- Doll et al. (2025) used scRNA-seq to identify regulators of cell death in the maize endosperm.
- van Weringh et al. (2025) used scRNA-seq to characterize drought responses in guard cells.

## Data Availability

No original data were generated for this *In Brief.*

## Dive Curated Terms

The following phenotypic, genotypic, and functional terms are of significance to the work described in this paper:

VND7 Gramene: At1g71930
VND7 Araport: At1g71930

VND6 Gramene: At5g62380
VND6 Araport: At5g62380
